# Playing Peekaboo with a Master Manipulator: Metagenetic Detection and Phylogenetic Analysis of *Wolbachia* Supergroups in Freshwater Invertebrates

**DOI:** 10.3390/ijms24119400

**Published:** 2023-05-28

**Authors:** Monika Mioduchowska, Edyta Konecka, Bartłomiej Gołdyn, Tom Pinceel, Luc Brendonck, Dunja Lukić, Łukasz Kaczmarek, Tadeusz Namiotko, Katarzyna Zając, Tadeusz Zając, Jan P. Jastrzębski, Krzysztof Bartoszek

**Affiliations:** 1 Department of Evolutionary Genetics and Biosystematics, Faculty of Biology, University of Gdansk, 80-308 Gdańsk, Poland; tadeusz.namiotko@ug.edu.pl; 2 Department of Invertebrate Zoology and Hydrobiology, Faculty of Biology and Environmental Protection, University of Lodz, 90-237 Łódź, Poland; 3 Department of Marine Plankton Research, Institute of Oceanography, University of Gdansk, 81-378 Gdynia, Poland; 4 Department of Microbiology, Institute of Experimental Biology, Faculty of Biology, Adam Mickiewicz University in Poznan, 61-614 Poznań, Poland; edyta.konecka@amu.edu.pl; 5 Department of General Zoology, Institute of Environmental Biology, Faculty of Biology, Adam Mickiewicz University in Poznan, 61-614 Poznań, Poland; bartlomiej.goldyn@amu.edu.pl; 6 Animal Ecology, Global Change and Sustainable Development, KU Leuven, 3000 Leuven, Belgium; tom.pinceel@kuleuven.be (T.P.); luc.brendonck@kuleuven.be (L.B.); 7 Centre for Environmental Management, University of the Free State, Potchefstroom 2520, South Africa; 8 Community Ecology Laboratory, Department of Biology, Vrije Universiteit Brussel (VUB), 1050 Brussels, Belgium; 9 Water Research Group, Unit for Environmental Sciences and Management, North-West University, Potchefstroom 2531, South Africa; 10 Department of Wetland Ecology, Estación Biológica de Doñana-CSIC, 41092 Sevilla, Spain; dunjalkc@gmail.com; 11 Department of Animal Taxonomy and Ecology, Faculty of Biology, Adam Mickiewicz University in Poznan, 61-614 Poznań, Poland; kaczmar@amu.edu.pl; 12 Institute of Nature Conservation, Polish Academy of Sciences, 31-120 Kraków, Poland; kzajac@iop.krakow.pl (K.Z.); tzajac@iop.krakow.pl (T.Z.); 13 Department of Plant Physiology, Genetics and Biotechnology, Faculty of Biology and Biotechnology, University of Warmia and Mazury in Olsztyn, 10-719 Olsztyn, Poland; bioinformatyka@gmail.com; 14 Genetics and Biotechnology, University of Warmia and Mazury in Olsztyn, 10-719 Olsztyn, Poland; 15 Department of Computer and Information Science, Division of Statistics and Machine Learning, Linköping University, SE-581 83 Linköping, Sweden

**Keywords:** Crustacea, Bivalvia, Tardigrada, next-generation sequencing, microbiome communities, *Wolbachia* endosymbiont

## Abstract

The infamous “master manipulators”—intracellular bacteria of the genus *Wolbachia*—infect a broad range of phylogenetically diverse invertebrate hosts in terrestrial ecosystems. *Wolbachia* has an important impact on the ecology and evolution of their host with documented effects including induced parthenogenesis, male killing, feminization, and cytoplasmic incompatibility. Nonetheless, data on *Wolbachia* infections in non-terrestrial invertebrates are scarce. Sampling bias and methodological limitations are some of the reasons limiting the detection of these bacteria in aquatic organisms. In this study, we present a new metagenetic method for detecting the co-occurrence of different *Wolbachia* strains in freshwater invertebrates host species, i.e., freshwater Arthropoda (Crustacea), Mollusca (Bivalvia), and water bears (Tardigrada) by applying NGS primers designed by us and a Python script that allows the identification of *Wolbachia* target sequences from the microbiome communities. We also compare the results obtained using the commonly applied NGS primers and the Sanger sequencing approach. Finally, we describe three supergroups of *Wolbachia*: (i) a new supergroup V identified in Crustacea and Bivalvia hosts; (ii) supergroup A identified in Crustacea, Bivalvia, and Eutardigrada hosts, and (iii) supergroup E infection in the Crustacea host microbiome community.

## 1. Introduction

Bacterial endosymbionts play an important role in many aspects of host ecology, metabolism, defense against pathogens, nutrition, reproduction, and evolution [1]. The best known intracellular bacterium on Earth is *Wolbachia*, belonging to the α-Proteobacteria [2]. It was first described as *Rickettsia*-like organisms (RLO) infecting gonad cells of the mosquito *Culex pipiens* Linnaeus, in 1758 and formally named *Wolbachia pipientis* Hertig and Wolbach, in 1924. Since its discovery in 1924 [3], this bacterial endosymbiont has intrigued biologists due to its significant impact on the evolution and reproductive biology, as well as the ecology of host species [4].

Currently, the highly genetically diversified genus *Wolbachia* is divided into 19 supergroups (monophyletic clusters), named with letters A to T, excluding G and R (Table 1). It is estimated that up to 76% of all terrestrial insect species are infected with this endosymbiont [5]. On the contrary, the occurrence of *Wolbachia* in aquatic invertebrates is much less studied and it was even hypothesized that *Wolbachia* infection had not reached aquatic environments [6]. However, data on the absence of *Wolbachia* in aquatic organisms are comparably scarce, thus the observed pattern may simply result from a sampling bias or methodological limitations. Nonetheless, to date, widespread *Wolbachia* infection in terrestrial invertebrates has been hypothesized to be most likely caused by the continental origin of this endosymbiont [7].

Detection of *Wolbachia* infections in non-terrestrial invertebrates is crucial for understanding the ecology and evolution of host species [32]. Interestingly, the occurrence of this endosymbiont in aquatic invertebrates is much more widespread than previously thought. In 1995, Sironi et al. [33] found a *Wolbachia*-like organism in Nematoda for the very first time. In 2018–2021, our team discovered *Wolbachia* infections in Tardigrada [34], Bivalvia [35] and Crustacea [32]. Later, Tibbs-Cortes et al. [36] also confirmed *Wolbachia* infection in the microbiome of Tardigrada. This allows us to suspect that more extensive sequencing would bring more new data of infections in new host taxa, potentially revising our understanding of *Wolbachia* evolution.

Unfortunately, molecular detection of *Wolbachia* is challenging. Difficulties in identifying these bacteria may arise from (A) the variable infection rate or prevalence in host species, i.e., *Wolbachia* strains may infect only a fraction of the host population [37]; (B) the inability to culture the bacteria on cell-free media and necessity to maintain them in hosts or cell lines [38]; (C) the co-infections of genetically different bacterial strains in the same individual [39]; (D) the horizontal gene transfer from *Wolbachia* to the host genome [40]; and (E) the insufficient titer of *Wolbachia* preventing successful detection [41].

Overall, in our previous research [32], the Sanger sequencing method failed to detect the presence of multiple *Wolbachia* infections in a single specimen of aquatic invertebrates. Since Sanger sequencing generally detects only the main PCR product, the application of this classical sequencing methodology does not allow the detection of coinfections with different *Wolbachia* strains showing multiple peaks on the chromatograms [42]. Fortunately, some of these obstacles can be solved by applying the next-generation sequencing (NGS) approach. In contrast to the Sanger sequencing method (e.g., Multi Locus Sequencing Typing—MLST), this technique is effective in detecting low-density infections and multiple strains occurring within the same host [43]. Therefore, in the present study we focus on four objectives: (i) to develop a new metagenetic method allowing the detection of the co-occurrence of different *Wolbachia* strains; (ii) to compare the results obtained using our primers, commercial NGS primers, and Sanger sequencing; (iii) to detect *Wolbachia* infection in aquatic invertebrates host species, i.e., water bears (Tardigrada), freshwater Arthropoda and Mollusca; and (iv) to classify the identified *Wolbachia* 16S rRNA sequences based on phylogenetic reconstruction of all supergroup strains.

## 2. Results

In Table 2, we provide the results of Sanger sequencing using our WOLBSR and WOLBSL primers, i.e., unreadable sequences obtained despite good quality PCR products, and the *Wolbachia* 16S rRNA gene fragment obtained using this sequencing approach in our previous studies [32]. In the present study, applying the existing WF and WR primers (annealing temperature 48 °C; [44]), we were the first to discover *Wolbachia* infection in *Eulimnadia* sp. (genus belonging to Branchiopoda; the sequence has been deposited in GenBank under accession number MZ901361).

High-throughput DNA sequencing of the V3-V4 region of the 16S rRNA gene using standard, commercial primers (341F and 785R) for all species yielded 94,269 to 247,184 high-quality reads binned into 53 to 2141 operational taxonomic units (OTUs) (Tables S1–S4). Using our primers (WOLBSR and WOLBSL) high-quality reads were obtained for 29% of the samples, i.e., from 212,122 to 289,746 reads and from 679 to 1427 OTUs, which allowed the microbiome analysis (multiple peaks on chromatograms based on Sanger sequencing were observed). The trace sequences were obtained for 57% of the samples, i.e., from 14 to 566, and the number of OTUs was from 7 to 92; thus it was not possible to analyze the microbiome communities of these samples (multiple peaks on chromatograms based on Sanger sequencing were observed). *Wolbachia* sequences for the remaining 14% of the samples were obtained using our primers and Sanger sequencing, and the NGS methodology was applied only using commercial primers ([32]; for details see Table 2).

**Table 2:** Summary of data generation and characterization of the 16S rRNA metagenetic library. Symbols: *—unreadable sequences obtained via Sanger sequencing using our designed primers, despite a good quality of PCR products; **—*Wolbachia* 16S rRNA gene fragment obtained via Sanger sequencing using our designed primers; ***—*Wolbachia* 16S rRNA gene fragment obtained via Sanger sequencing using *Wolbachia*-specific WF and WR primers.

Phylum	Species/Genus (Isolate ID)	Sequence Count		No of Observed OTUs	
		Result with Commercial 341F/785R Primers (NCBI SubmissionID, Source)	Result with Our Designed WOLBSR/WOLBSL Primers (NCBI SubmissionID, Source)	Result with Commercial 341F/785R Primers	Result with Our Designed WOLBSR/WOLBSL Primers
**ARTHROPODA**	*Artemia salina* (AS)	219060 (SUB10812623, our study)	274882 (SUB10815753, our study) *	690	270
	*Artemia* *parthenogenetica* (AP)	229518 (SUB10812623, our study)	212122 (SUB10815753, our study) *	679	671
	*Branchipus* *schaefferi* (PA)	247184 (SUB10812623, our study)	16S rRNA obtained by Sanger sequencing (GenBank: MH447361, [32]) **	945	NA
	*Branchipus* *schaefferi* (SRB1)	212948 (SUB10812623, our study)	122 (SUB10815753, our study) *	946	40
	*Chydorus* sp. (ALTAJ2)	125306 (SUB10812623, our study)	566 (SUB10815753, our study) *	395	92
	*Eulimnadia* sp. (CON)	215478 (SUB10812623, our study)	265656 (SUB10815753, our study) *, 16S rRNA obtained by Sanger sequencing (GenBank: MZ901361) ***	1171	1427
	*Streptocephalus* *cafer* (SC)	220824 (SAMN13134284, [34])	16S rRNA obtained by Sanger sequencing (GenBank: MH447357, [32]) **	400	NA
	*Triops* *cancriformis* (TCO)	129736 (SUB10812623, our study)	290 (SUB10815753, our study) *	1311	44
**MOLLUSCA**	*Unio crassus* (C3Nf)	94269 (SUB10812623, our study)	120 (SUB10815753, our study) *	53	50
	*Unio crassus* (P3Nf)	117498 (SUB10812623, our study)	76 (SUB10815753, our study) *	87	34
	*Dreissena* *polymorpha* (RAC)	220716 (SUB10812623, our study)	289746 (SUB10815753, our study) *	2141	1253
**TARDIGRADA**	*Paramacrobiotus experimentalis* (MAD-TAR9)	213744 (PRJNA530068, [45])	14 (SUB10815753, our study) *	359	7
	*Paramacrobiotus experimentalis* (MAD-TAR11)	185330 (PRJNA530068, [45])	38 (SUB10815753, our study) *	351	15
	*Macrobiotus* *basiatus* (8aUSA)	199186 (SUB10812623, our study)	130 (SUB10815753, our study) *	610	57

**Figure 1:**
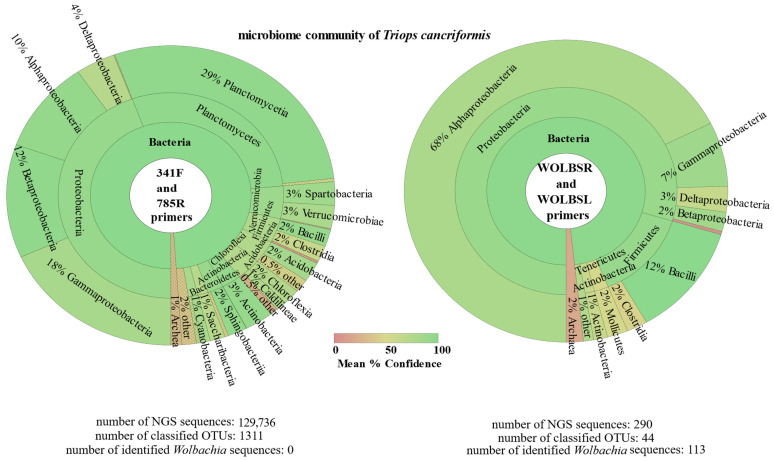
Comparison of the *Triops cancriformis* microbiome community using commercial (341F and 185R) and designed (WOLBSR and WOLBSL) NGS primers.

**Figure 2:**
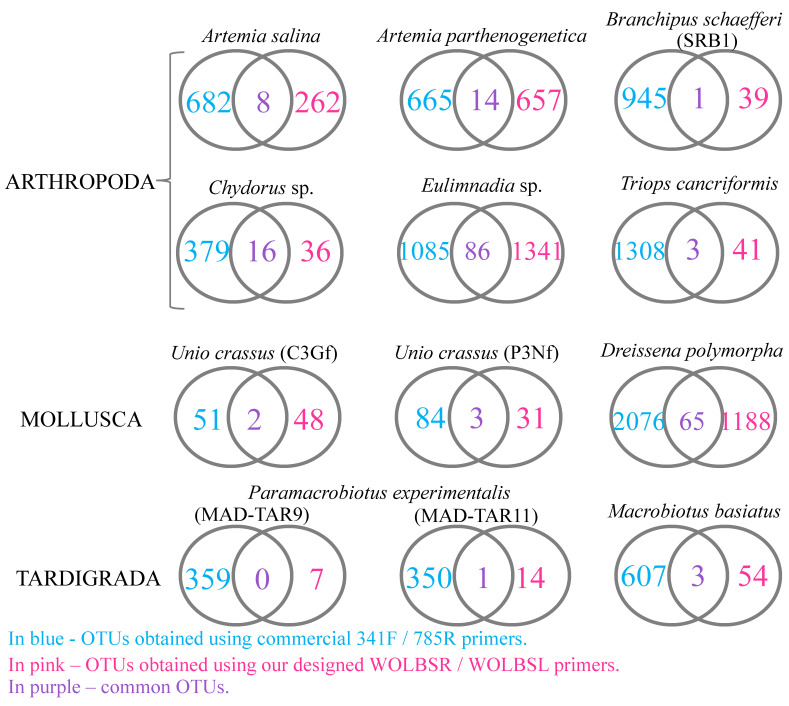
Venn diagram illustrating the core and specific microbiome OTUs of all samples.

Our results underscore the trade-off between the sensitivity and specificity of individual primer sets. The commercial primers were more sensitive to widespread bacterial species, but had almost no specificity for *Wolbachia* sequences. On the other hand, our primers improved *Wolbachia* detection and were more sensitive, qualifying them for more extensive tests conducted on the microbiome communities (Table 3; for an example comparing microbiome profiles of *Triops cancriformis* see Figure 1). As a result, when using the two different primer sets, no more than 7% of the OTUs were common for the individual host of the same species (Figure 2).

**Figure 3:**
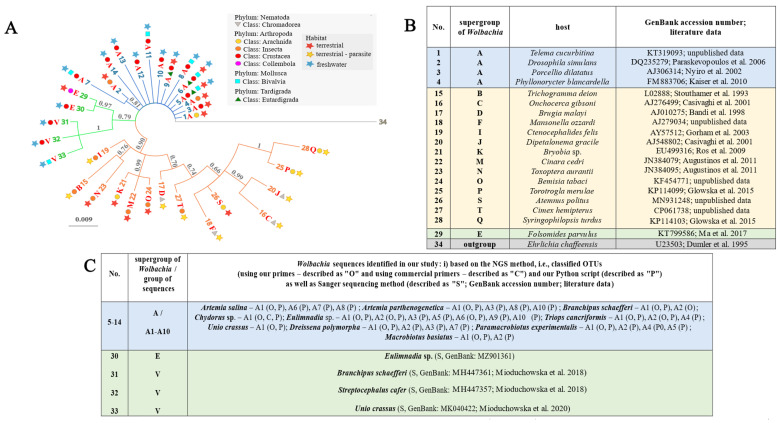
(**A**) The Bayesian tree of *Wolbachia* supergroups based on newly obtained 16S rRNA gene sequences and strains downloaded from the GenBank database. Phylogenetic reconstructions conducted using the HKY+G as the best-fitting model of evolution. Values of posterior probabilities (PP) are presented above the branches (nodes with PP < 0.60 were collapsed). (**B**) The list of *Wolbachia* sequences and outgroup downloaded from the GenBank database. (**C**) The list of *Wolbachia* sequences obtained in the present and our previous works. Citations that appeared in the Figure: Ma et al. 2017 [15]; Gorham et al. [22]; Ros et al. 2009 [23]; Augustinos et al. 2011 [25]; Glowska et al. 2015 [28]; Mioduchowska et al. 2018 [32]; Mioduchowska et al. 2020 [35]; Paraskevopoulos et al. 2006 [48]; Nyiro et al. 2002 [49]; Kaiser et al. 2010 [50]; Stouthamer et al. 1993 [51]; Casivaghi et al. 2001 [52]; Bandi et al. 1998 [53]; Dumler et al. 1995 [54].

We screened the GenBank database for target *Wolbachia* sequences from the microbiome communities (for workflow details see Figure 6 in Section 4). The Hamming distance for all of our 16S rRNA sequences identified as *Wolbachia* endosymbionts did not exceed 0.07 (Table 3 and Tables S5–S16). We also combined all available information to reconstruct the phylogenetic relationships between all *Wolbachia* supergroups, resulting in classifying our sequences into supergroup A and supergroup E, and a new supergroup V (Figure 3). Interestingly, we identified three main clades consisting of (i) the oldest and very diverse supergroup A found in hosts inhabiting terrestrial and freshwater environments, i.e., Arachnida, Insecta, Crustacea, Bivalvia, and Eutardigrada (clade marked in blue in Figure 3); (ii) supergroup E identified in a Collembola host from terrestrial habitat, a Crustacea host from freshwater habitat, and the new supergroup V found in hosts inhabiting only freshwater habitats (Crustacea and Bivalvia) (clade marked in green in Figure 3); and (iii) the youngest and more deeply subdivided clade grouping all other previously identified *Wolbachia* supergroups (I, B, N, K, M, O, D, T, F, S, C, J, P, and Q) infecting terrestrial hosts (including parasitic species), i.e., Nematoda, Arachnida, Insecta, and Crustacea (clade marked in orange in Figure 3).

The ranges of uncorrected genetic p-distances between *Wolbachia* supergroups obtained in our study and all other supergroups of *Wolbachia* were as follows:

(i) the supergroup A: no differences were found between our group of sequences described as the A1 supergroup of *Wolbachia* and the A supergroup of *Wolbachia* identified in *Telema cucurbitina* Wang, Chunxia and Li 2010 ([46], Arachnida; GenBank accession number: KT319093; however, these *Wolbachia* sequences were previously wrongly classified to the R supergroup), and the least similar was (comparing with the A8 supergroup of *Wolbachia*) the C supergroup of *Wolbachia* found in *Onchocerca gibsoni* Cleland and Johnston, 1910 ([47], Nematoda; GenBank accession number: AJ276499) with a genetic distance value of 6%; 

(ii) the supergroup E: no differences were detected compared to this strain identified in *Folsomides parvulus* Stach, 1922 (Collembola; GenBank accession number: KT799586), and the least similar was the T supergroup of *Wolbachia* infection found in *Cimex hemipterus* (Fabricius, 1803) ([29], Insecta; GenBank accession number: CP061738) with a genetic distance value of 3.7%; 

(iii) the supergroup V: the most similar was the E supergroup of *Wolbachia* found in our study (host: *Eulimnadia* sp.; Crustacea) and previously described infection in *F. parvulus* with a genetic distance value of 1.8%, and the least similar was the C supergroup of *Wolbachia* identified in *O. gibsoni* with a genetic distance value of 6.4% (Table S17).

Applying the metagenetic approach allowed to identify different OTUs belonging to the A supergroup of *Wolbachia* (Figure 3). 

Only a single OTU (group of sequences described as A1) of this diverse supergroup was found in the following freshwater invertebrates:

- *Unio crassus* (isolate ID: C3Nf);
- *Chydorus* sp. (isolate ID: ALTAJ2).

Multiple coinfections of different groups of sequences from the A supergroup of *Wolbachia*, i.e., from A1 to A10, were identified in the following organisms:

- *Artemia salina* (isolate ID: AS)—four groups of sequences (A1, A6, A7, and A8);
- *A. parthenogenetica* (isolate ID: AP)—four groups of sequences (A1, A3, A8, and A10);
- *Branchipus schaefferi* (isolate ID: SRB1)—two groups of sequences (A1, and A2);
- *Eulimnadia* sp. (isolate ID: CON)—seven groups of sequences (A1, A2, A3, A5, A6, A9, and A10);
- *Triops cancriformis* (isolate ID: TCO)—three groups of sequences (A1, A2, and A4);
- *Dreissena polymorpha* (isolate ID: RAC)—four groups of sequences (A1, A2, A3, and A7);
- *Paramacrobiotus experimentalis* (isolate ID: MAD-TAR9)—two groups of sequences (A1, and A2);
- *Pam. experimentalis* (isolate ID: MAD-TAR9)—four groups of sequences (A1, A2, A4, and A5);
- *Macrobiotus basiatus* (isolate ID: 8aUSA)—two groups of sequences (A1, and A2).

In turn, the Sanger sequencing method allowed to find a single infection of the V supergroup in the following:

- *B. schaefferi* (isolate ID: PA);
- *Streptocephalus cafer* (isolate ID: SC).

Moreover, the Sanger sequencing approach was useful to identify additional supergroups infections in the following:

- *Eulimnadia* sp. (isolate ID: CON)—the E supergroup;
- *U. crassus* (isolate ID: C3Nf)—the V supergroup.

**Figure 4:**
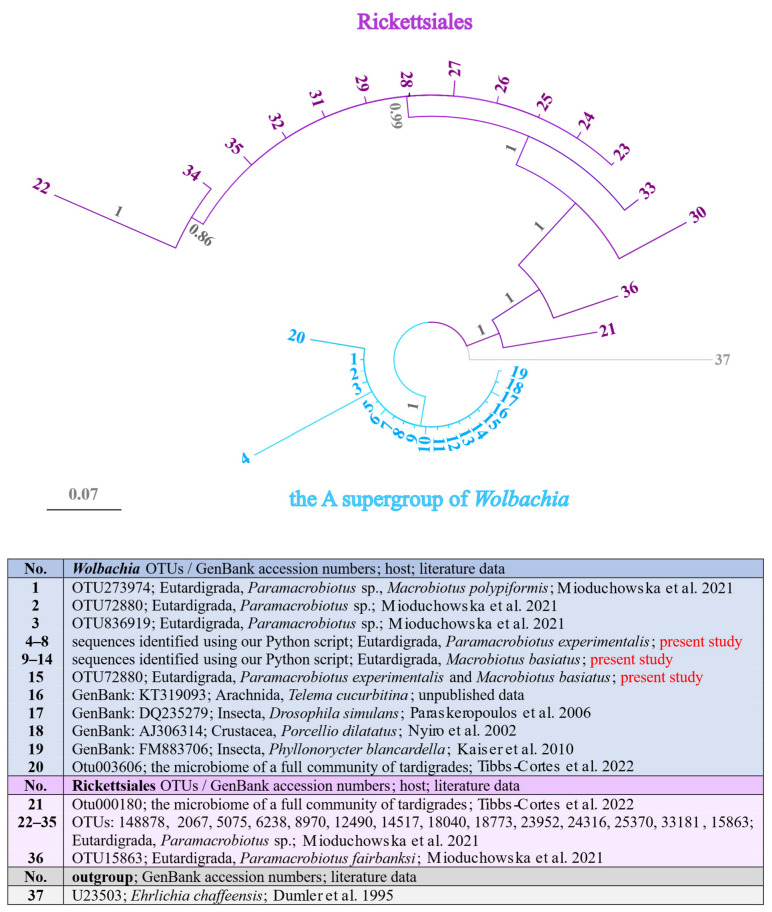
The Bayesian tree of *Wolbachia* and Rickettsiales 16S rRNA gene sequences identified in Tardigrada, and previously described host species downloaded from the GenBank database. Phylogenetic reconstructions conducted using the HKY+G as the best-fitting model of evolution. Values of posterior probabilities (PP) are presented above the branches (nodes with PP < 0.60 were collapsed). Citations that appeared in the Figure: Mioduchowska et al. 2021 [34]; Tibbs-Cortes et al. 2022 [36]; Paraskevopoulos et al. 2006 [48]; Nyiro et al. 2002 [49]; Kaiser et al. 2010 [50]; Dumler et al. 1995 [54].

To check whether all *Wolbachia* infections found in Tardigrada represented the same supergroup, we carried out the phylogenetic reconstructions of tardigrade *Wolbachia* 16S rRNA sequences identified in our study and in previous surveys [34,36]. All of these sequences were also compared with other Rickettsiales sequences obtained by Mioduchowska et al. [34] and Tibbs-Cortes et al. [36]. Reconstructed phylogenetic relationships positioned our new sequences together with *Wolbachia* sequences from previous studies in a clade consisting of strains belonging to the supergroup A (Figure 4). The second clade consisted of sequences that originated from other species belonging to other Rickettsiales. The genetic p-distance value calculated for the first clade consisting of the A supergroup of *Wolbachia* was 0% to 1.8% (0.6% on average). The uncorrected genetic p-distances between our sequences described as supergroup A and the other sequences of the Rickettsiales ranged from 17.1% to 33.7% (23.6% on average) (Table S18).

*Wolbachia* infections in other freshwater invertebrates, i.e., Branchiopoda and Bivalvia, were detected for the first time in our study; therefore, phylogenetic reconstructions of *Wolbachia* 16S rRNA sequences within these taxonomic groups were not possible.

## 3. Discussion

### 3.1. General Remarks

Detection of *Wolbachia* is complex when different strains occur in low frequencies in the host microbiome community and their abundance prevents obtaining good quality sequences using the Sanger method. In fact, various *Wolbachia* strains infect many Arthropoda [55], which implies that frequent coinfections are likely and common. As a result, the presence of *Wolbachia* in freshwater invertebrates could have been previously missed due to the sampling bias and the lack of suitable bioinformatic tools or PCR primers for molecular marker amplification. To overcome this problem, we present a new metagenetic method allowing the detection of the co-occurrence of different *Wolbachia* strains.

We performed a high-throughput sequencing based on the hypervariable V3-V4 region of the bacterial 16S rRNA gene using both non-degenerate primers designed by us and the other commercial primers. Additionally, we have also applied the Sanger sequencing approach. Overall, the 16S rRNA gene is a good tool for *Wolbachia* phylogenetic studies, as it contains hypervariable regions that enable the identification of phylogenetic differences between microorganisms. The V4 region is associated with the shortest geodesic distance, which implies that it may be the optimal choice for phylogeny-related studies, including phylogenetic analysis of novel *Wolbachia* supergroups. The V3 region has a high resolution for bacterial phyla and is useful for studying bacterial diversity in various environments. It allows for a more precise distance-based clustering of reads into phyla-level OTUs. Moreover, for short-amplicon sequencing, a literature survey has shown that the V3-V4 region is the most commonly applied in phylogenetic analyses of various bacteria (e.g., [56]).

We focused on three taxonomic groups, i.e., freshwater Arthropoda (Crustacea), Mollusca (Bivalvia) and water bears (Tardigrada), with known and unknown *Wolbachia* infection status. Metagenetic microbiome analysis allowed us to indicate that our primers were more sensitive and specific for detecting *Wolbachia* in freshwater invertebrates, in contrast to the results obtained using the widely applied commercial primers and the Sanger sequencing approach. We also introduced a new Python script and bioinformatics pathway for identifying target *Wolbachia* sequences from the microbiome communities based on Hamming distance values. Finally, based on the phylogenetic relationships of all *Wolbachia* supergroups available in public databases, we detected three supergroups of *Wolbachia*: (i) a new supergroup V identified in Crustacea and Bivalvia hosts; (ii) supergroup E infection in Crustacea host microbiome community; and (iii) diverse supergroup A identified in Crustacea, Bivalvia, and Eutardigrada hosts. 

### 3.2. Wolbachia in Freshwater Arthropods with Special Emphasis on Crustacea

Sazama et al. [57] conducted a global review of the incidence of *Wolbachia* in 228 species of aquatic insects based on the 16S rRNA marker and estimated that 52% of the tested species were infected by *Wolbachia*. The incidence of these bacteria was common among aquatic insects, but the level of infection differed considerably between orders. In most cases, however, only a minority (<10%) of individuals within species were infected [58]. Although *Wolbachia* is common among terrestrial and marine isopods, it has rarely been detected in other Crustacean groups, including those inhabiting freshwater environments [7]. Only one species of Isopoda [7], two species of Branchiopoda [32], and four species of Copepoda [59] were so far known to be the hosts of *Wolbachia*. 

In the present study, we confirmed the infection of *Wolbachia* in seven freshwater Crustacea hosts, including two Anostraca (*B. schaefferi* and *S. cafer*), previously found to be infected with *Wolbachia* using the Sanger sequencing [32]. For the remaining four species, i.e., *A. salina*, *A. parthenogenetica*, *Eulimnadia* sp., and *T. cancriformis*, *Wolbachia* was detected via NGS only using primers designed by us, while for *Chydorus* sp. parallel analyses using both commercial and NGS primers designed by us succeeded, although with a very low infection rate. Overall, *Wolbachia* infections identified using our primers ranged from 0.01% (*A. parthenogenetica*) to 38.97% (*T. cancriformis*) of the total microbiome community (Table 3). 

The phylogenetic analysis of *Wolbachia* showed the following infection pattern in all studied species (Figure 3, Table S17):

(i) supergroup A based on NGS approach (described here as subgroups A1–A10), widely identified in numerous Arthropoda including Insecta, Arachnida, and Isopoda, where *Wolbachia* exhibits host mutualism and causes reproductive parasitism effect [11];

(ii) supergroup E identified in *Eulimnadia* sp. based on Sanger sequencing, found also in Collembola [17,18], where it demonstrated mutualistic relations with the host [60] and causing reproductive parasitism [61]; and

(iii) a new supergroup V found in *S. cafer* and *B. schaefferi* based on the Sanger sequencing approach (primers by [32]).

### 3.3. Wolbachia in Freshwater Bivalvia

The problem of endosymbionts causing infectious diseases with massive mortality rates has long been considered of significant economic value in marine bivalves, such as oysters or scallops [62]. They were initially identified as RLO based on the ultrastructural features. Interestingly, Cruz-Flores and Caceres-Martinez [63] have reported in a recent review that RLO appear to be symbionts of more than 60 species of Bivalvia, marine Gastropoda of aquacultural importance worldwide, and one freshwater alien invasive species *Dreissena* sp.. We cannot exclude the possibility that these infections were actually caused by *Wolbachia*(-like) bacteria [64]. The difficulties seem to be addressed with the use of the genetic approach in the 1990′s to facilitate the identification of alternative hosts for RLO and its modes of transmission [65]. In 1998, Schilthuizen and Gittenberger [66] screened 38 species of Mollusca (24 terrestrial Gastropoda, 11 freshwater Gastropoda, and 3 freshwater Bivalvia) and found no *Wolbachia* infections. Subsequently, several authors have cited this paper, suggesting that *Wolbachia* is not present in Mollusca [67] and stressing the need for further research on infections in this group [68].

The only *Wolbachia* sequences in bivalves were those reported by Mioduchowska et al. [35] for *U. crassus*. These sequences have been clustered in a clade containing a possible new strain, which is here named supergroup V. In this study, *Wolbachia* was rediscovered in *U. crassus* (previously reported in [35]) and was discovered for the first time in *D. polymorpha*. Both infections were detected using our designed primers. Phylogenetic analysis revealed infection with various strains belonging to the supergroup A, described here as subgroups A1–A4 and A7 (Figure 3, Table S17). It should be emphasized that the presented results of *Wolbachia* infections in freshwater Mollusca are completely novel. 

### 3.4. Wolbachia in Tardigrada

The current state of knowledge on the microbial communities associated with Tardigrada is very limited. In 2018, Vecchi et al. [69] showed the presence of taxon-specific symbionts that largely contributed to the identification of differences in microbiome profiles between Tardigrada species. The bacterial order Rickettsiales has also been shown to be common in all Tardigrada studied [69]. However, OTUs associated with putative *Wolbachia* endosymbionts have not been identified. By using an entire mount of fluorescent in situ hybridization (FISH) in the parthenogenetic heterotardigrade *Echiniscus trisetosus* Cuénot, (1932) [70,71], a putative bacterial endosymbiont has been detected within the ovary of a parthenogenetic population, indicating a possible maternal transmission from mother to offspring. At the same time, Kaczmarek et al. [45] identified two OTUs belonging to a putative bacterial endosymbiont of Rickettsiales in eutardigrade *Pam. experimentalis*. In 2020, Guidetti et al. [71] observed four putative endosymbionts of Tardigrada from the group of α-Proteobacteria, which were classified into the same larger clade as *Wolbachia*.

This is the third study that confirms the presence of putative endosymbionts in Tardigrada. In 2021 and 2022, Mioduchowska et al. [34] and Tibbs-Cortes et al. [36], respectively, identified *Wolbachia* lineages based on high-throughput sequencing of the 16S rRNA bacterial gene. In the current survey, *Wolbachia* was found in both investigated water bear species. In the case of *Pam. experimentalis,* only designed primers gave a positive signal. In turn, in *Mac. basiatus* infection was found using both commercial and designed primers. Phylogenetic analyses revealed infection with various strains belonging to the supergroup A, described here as subgroups A1–A2 and A4–A5 (Figure 3, Table S17). This approach also confirmed that despite the low frequency of *Wolbachia* in the Tardigrada microbiome community, these strains always clustered in supergroup A (Figure 4, Table S18).

We identified a putative tardigrade *Wolbachia* endosymbiont at low relative abundance in the microbiome community (Table 3), and these findings were consistent with the data reported by Mioduchowska et al. [34] and Tibbs-Cortes et al. [36]. Such a low prevalence of *Wolbachia* has been previously reported in Arthropoda [72]; however, whether and how such infection is maintained in hosts remain an open question. Nevertheless, considering that *Wolbachia* generally seems to occur at very low frequencies, Sanger sequencing methods could have failed to detect the infections [32,33].

### 3.5. Future Research Prospects: Losing or Winning with the Master Manipulator?

*Wolbachia* bacteria interact with their hosts through parasitic manipulation of the reproductive system as a secondary endosymbiont and mutually as a primary endosymbiont [73]. This bacterial endosymbiont can also provide benefits to hosts [74]. In all the described associations, *Wolbachia* can be transmitted horizontally (resulting in the lack of co-speciation in Arthropoda), vertically (congruence of *Wolbachia* and filarial phylogenies of Nematoda), or both [75]. As a rule, it is transmitted through the female germ-line cells to the offspring [76]. In addition to the germ-line cells, it is known that a range of other somatic tissues can also be infected [77]. Moreover, horizontal transmission of *Wolbachia* between phylogenetically close and distant hosts, or directly from the environment, has been detected in many cases [78]. Thus, *Wolbachia* was considered to occur in non-arthropod hosts. Multiple infections of a very diverse supergroup A have been found in all taxonomic groups in our study. We suggest that such an occurrence of closely related *Wolbachia* strains in phylogenetically distant invertebrate lineages may be well explained by a widespread horizontal transfer. To date, multiple mechanisms of *Wolbachia* transmission have been proposed, but the factors influencing *Wolbachia* transmission into new hosts are still poorly understood [79]. Nonetheless, the evolved modes of transmission between host species in water bears and freshwater mussels as well as freshwater Arthropoda should be investigated. We cannot rule out the possibility that supergroup E and new supergroup V can be transmitted vertically, which is believed to be the dominant mode of transmission of *Wolbachia* between hosts (Figure 3; [80]).

In the course of evolution, *Wolbachia* could have induced several reproductive phenotypes in their hosts, including feminization, early and late male-killing, parthenogenesis, and cytoplasmic incompatibility [76]. As of 2010, the question “May parthenogenesis in *Artemia* be attributed to *Wolbachia*?” [81] has been still open. The authors screened parthenogenetic and bisexual *Artemia* sp. populations from all over the world for *Wolbachia*, using the 16S rRNA gene fragment and Sanger sequencing. As in our previous studies [32], they obtained weak sequences or other bacterial species from PCR products of good quality. Finally, Maniatsi et al. [81] concluded that *Artemia* sp. was rather unlikely to be the host of *Wolbachia*, and therefore parthenogenesis could not be induced by this endosymbiont. Contrary to that, we did discover *Wolbachia* infection in *Artemia* sp.; however, it remains to be investigated if parthenogenesis is really connected with *Wolbachia* infections in this crustacean species.

The symbiosis between *Wolbachia* and the host can be beneficial to both partners. The bacterial endosymbionts provide vitamins B to enhance reproduction of the hosts [82] and to strengthen their fecundity. In turn, benefits to bacteria are rarely measured [83]. It is noteworthy that among the identified *Wolbachia* strains of the supergroup A, the genes involved in stress resistance and modulation of host cell functions have been discovered, whereas the ankyrin repeat (ANK) containing genes have been identified in the supergroup E, and according to Faddeeva-Vakhrusheva et al. [84], these genes play a role in the feminization process. Nevertheless, thus far specific gene functions in the newly discovered supergroup V remain unidentified. It is worthwhile to mention that *Wolbachia* can reduce pathogenic viral loads in various arthropods [85]. It has an ability to limit disease transmission (e.g., Zika, dengue, chikungunya as well as malaria) not only by reducing the number of infectious mosquitoes in a population, but also by delaying the arrival of virus in the saliva [86]. 

The *Wolbachia* infections presented in our study may be conserved by the new host. Overall, since we know almost nothing on the dominance of the *Wolbachia* infection in freshwater invertebrates, its abundance could be different than that found in terrestrial Arthropoda. Moreover, since the data on *Wolbachia* presence in freshwater hosts are very scarce, it is hypothesized that the widespread colonization of terrestrial Arthropoda by this endosymbiont could be caused by its continental origin [7]. However, this hypothesis is questionable, since according to our results the oldest clades are common in species inhabiting terrestrial and freshwater environments, while the youngest clade consists of only terrestrial (mostly parasitic) species. Therefore, future research should focus on detecting *Wolbachia* infections in other freshwater invertebrate species, as well as on the ecological and evolutionary relationships between the new host species and the “master manipulator”. Last but not least, future research should also focus on the detection of infections at the cytogenetic level by incorporating the FISH technique to gain insight into the distribution of *Wolbachia* in various tissues. Since our current primers are not suitable for FISH applications, specific probes should be designed for this purpose. Our primers, on the other hand, allow the amplification of 16S rRNA sequences of the bacterial endosymbiont *Wolbachia*, as well as other (endo)symbionts [32] and members of the microbial community (present study). Therefore, the FISH approach will allow us to localize the 16S rRNA sequences of various bacteria, and not only the target sequences of *Wolbachia*. Finally, our findings open new frontiers in the *Wolbachia*-driven biology and ecology of the investigated invertebrates, and also confirm that the range of *Wolbachia* host species is significantly wider than previously thought. Moreover, the method described in the present study (including a new Python script) offers new perspectives for detecting multiple infections in a single host.

## 4. Materials and Methods

### 4.1. Sample Collection, Species Identification and DNA Extraction

Information on the data sets and sampling sites are presented in Table 4. The DNA of freshwater Arthropoda was acquired according to the procedures described by Mioduchowska et al. [32,87]. We used DNA isolates of Anostraca species: *Branchipus schaefferi* Fischer, 1834 [88] and *Streptocephalus cafer* (Lovén, 1847) [89] in which *Wolbachia* infection was discovered for the first time by Mioduchowska et al. [32] (see also [34] for more data on *S. cafer*). We also applied DNA isolates of other Branchiopoda, i.e., *Artemia salina* (Linnaeus, 1758) [90], *Artemia parthenogenetica* Bowen and Sterling, 1978 (sensu [91]), *Chydorus* sp., *Eulimnadia* sp., and *Triops cancriformis* (Bosc, 1801) [92]. Approximately 3 mm^3^ of thorax tissue was used to extract DNA from the selected species, with the exception of *Chydorus* sp. for which DNA extraction was performed from whole individuals, ca. 1 mm^3^. Molluscan isolates were extracted from two Bivalvia species, i.e., two *Unio crassus* (Philipsson, 1788) (sensu [93]) populations [gonad tissue from Czarna Hańcza River population (Poland)—previously recorded *Wolbachia* infection in the foot tissue] [35]; foot tissue from Pilica River population (Poland); samples of ca. 3 mm^3^ volume each] and *Dreissena polymorpha* (Pallas, 1771) [94] (from the whole body, ca. 5 mm^3^), following the methodology described by Mioduchowska et al. [35,95]. In the case of Tardigrada, two *Paramacrobiotus experimentalis* Kaczmarek, Mioduchowska, Poprawa and Roszkowska, 2020 isolates described by Kaczmarek et al. [45] were used. We also used a new Tardigrada species, i.e., *Macrobiotus basiatus* Nelson, Adkins Fletcher, Guidetti, Roszkowska, Grobys and Kaczmarek, 2020 isolate [96], which was obtained using the same extraction methodology as described for *Pam. experimentalis* [45]. Genomic DNA was extracted from entire tardigrade specimens using the protocol described by Mioduchowska et al. [34]. In total, 70 isolates, i.e., 5 isolates per population were used (Table 4).

All selected invertebrates were identified at the species/genus level based on integrative taxonomy, i.e., morphological criteria and the mitochondrial cytochrome oxidase subunit I (COI) gene sequences. The same DNA isolates as for the microbiome analysis were used. The barcode sequences of Crustacea, Bivalvia and Eutardigrada were amplified using the universal invertebrate primers: HCO2198 (5′-TAAACTTCAGGGTGACCAAAAAATCA-3′) and LCO1490 (5′-GGTCAACAAATCATAAAGATATTGG-3′) [97]. The PCR protocols described in our previous papers were applied as follows: (i) for species of Crustacea, the PCR parameters described by Lukić et al. [98]; (ii) for species of Bivalvia, protocol provided by Kilikowska et al. [99]; and (iii) for species of Eutardigrada, protocol according to Kaczmarek et al. [45]. In the case of *A. salina*, contaminations of COI sequences were obtained (as previously indicated in other anostracan species [87]). Consequently, to overcome this problem, more conservative molecular marker was applied, i.e., the fragment of the ribosomal 18S gene. The PCR amplifications were performed using eukaryote-specific primers: complementary to the 5′-terminus (5′-TYCCTGGTTGATYYTGCCAG-3′) and the 3′-terminus (5′-TGATCCTTCCGCAGGTTCACCT-3′) [100], with the PCR protocol provided by Mioduchowska et al. [101].

The obtained sequences were checked for the quality and manually aligned in the BioEdit ver. 7.2.5 [102]. The comparison of obtained sequences with GenBank records and the homology search was carried out with Basic Local Alignment Search Tool (BLAST, [103]) using blastn searches. All obtained sequences have been deposited in GenBank under the accession numbers provided in Table 4. Finally, only invertebrates with obtained barcode sequences were included in the present study. Some of the studied species, however, are marked as “sp.”, since these may represent species new to science awaiting formal descriptions. Genus abbreviations for Tardigrada follow Perry et al. [104].

All laboratory procedures were performed using sterile equipment and all steps were carried out in a sterile laminar flow hood to avoid cross-contamination of the samples. Moreover, the “RIDE” checklist, i.e., a set of minimal experimental criteria, to improve the reliability of samples with low microbial biomass (especially those obtained from Tardigrada) was applied [98]. When a blank template was applied (negative control), the resulting PCR products failed quality control tests for NGS analysis (using commercial 341F/785R primers—PCR products quality was too poor to perform high-throughput sequencing) or no visible PCR products were obtained (using WOLBSL/WOLBSR primers designed by us), confirming that there was no DNA contamination in the extraction reagents. The quality and quantity of extracted DNA were evaluated using a NanoDrop ND-1000 UV–Vis (Thermo Fisher Scientific). Then, the extracted genomic DNA was stored at −20 °C until further analyses.

**Table 4:** Summary of sampling species and data sets.

Phylum	Taxa (Isolate ID; GenBank Accession Number of Barcode Sequences, and Source)	Sources of Samples (Locality)
**ARTHROPODA**	*Artemia salina* (AS; GenBank: OL872292, our study)	the adults of *Artemia salina* acquired from IchthyoTrophic company (Poland)
	*Artemia parthenogenetica* (AP; GenBank: OL872290, our study)	the cysts of *Artemia parthenogenetica* acquired from Artemia Koral Gmbh company (Germany)
	*Branchipus schaefferi* (PA; GenBank: MK465076, [105])	provided by Lukić et al. [105] (Poland; Pila)
	*Branchipus schaefferi* (SRB1; GenBank: MK564494, [105])	provided by Lukiclet al. [105] (Serbia; Northern Banat)
	*Chydorus* sp. (ALTAJ2; GenBank: OL889759, our study)	provided by the project INERACT 730,938 H2020 attributed to T. Namiotko and S. Iepure (Russia; Altai Mts.)
	*Eulimnadia* sp. (CON; GenBank: OL889761, our study)	provided by the project of Univ. Gdansk 530-L155-D249-17/18 attributed to T. Namiotko (Mauritius; Rodrigues Island)
	*Streptocephalus cafer* (SC; GenBank: OL872295, our study)	provided by Mioduchowska et al. [32] (South Africa; locality described in paper as “Station 2”)
	*Triops cancriformis* (TCO; GenBank: OL872296, our study)	provided by Mioduchowska et al. [32] (Poland; locality described in paper as “Station 4”)
**MOLLUSCA**	*Unio crassus* (C3Gf; GenBank: OL872298, our study)	provided by Mioduchowska et al. [95] (Poland; Czarna Hańcza River)
	*Unio crassus* (P3Nf; GenBank: OL872299, our study)	provided by Mioduchowska et al. [95] (Poland; Pilica River)
	*Dreissena polymorpha* (RAC; GenBank: OL913806, our study)	collected from the Vistula drainage (52°37′04′′N, 19°19′42′′E)
**TARDIGRADA**	*Paramacrobiotus experimentalis* (MAD-TAR9; GenBank: MN097836, Kaczmarek et al. [45])	provided by Kaczmarek et al. [45] (the Toamasina and Antananarivo Provinces in Madagascar)
	*Paramacrobiotus experimentalis* (MAD-TAR11; GenBank: MN097837, Kaczmarek et al. [45])	provided by Kaczmarek el.al. [45] (the Toamasina and Antananarivo Provinces in Madagascar)
	*Macrobiotus basiatus* (8aUSA; GenBank: OL943796, our study)	provided by Nelson et al. [96] (the campus of East Tennessee State University, Johnson City, Tennessee)

### 4.2. Sanger Sequencing Approach

Preliminary detection of *Wolbachia* was performed using PCR screening and Sanger sequencing. We applied *Wolbachia*-specific WF (forward: 5′–CGGGGGAAAATTTATTGCT–3′) and WR (reverse: 5′–AGCTGTAATACAGAAAGGAAA–3′) primers according to the PCR protocol provided by Singh et al. [44], as well as our designed WOLBSL (forward: 5′–GCTAGTTGGTGGAGTAATAGCC–3′) and WOLBSR (reverse: 5′–GACTACCAGGGTATCTAATCCTG–3′) primers according to the PCR protocol described by Mioduchowska et al. [32]. The PCR reactions were performed in a BiometraTProfessional thermocycler. Amplified products were cleaned up via exonuclease I (20 U/μL, Thermo Scientific) and alkaline phosphatase FastAP (1 U/μL, Thermo Scientific): incubation at 37 °C for 15 min and heating at 85 °C for 15 min. The Sanger sequencing was carried out in both directions using the BigDyeTM terminator cycle sequencing and ABI Prism 3130xl Genetic Analyzer (Life Technologies). Obtained sequences were checked for quality and were manually aligned in BioEdit v. 7.2.5. The BLAST search tool searches were performed to verify the identity and homology of the amplified *Wolbachia* gene fragment with sequences deposited in the NCBI database. We accepted only the results which indicated query cover near 100%, high identity ˃95% and an E value near 0.0. All obtained sequences have been deposited in GenBank under the accession numbers provided in Table 4.

### 4.3. Designed vs. Commercial NGS Primers—Amplification of the Bacterial 16S rRNA Gene Fragment

**Figure 5:**
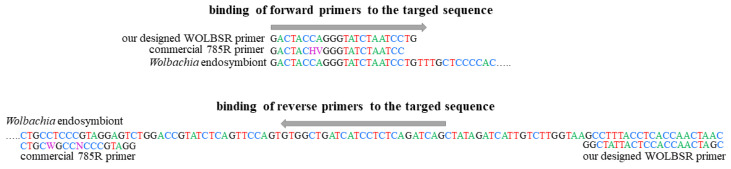
Annealing sites of the primers used to amplify the bacterial 16S rRNA gene fragment.

We performed high-throughput sequencing using the new isolates of invertebrates (i.e., representatives of the three freshwater invertebrate phyla, i.e., Arthropoda (Crustacea), Mollusca (Bivalvia), and water bears (Tardigrada)) and those described in our previous papers ([32,35,87,95,105]; Table 4). Metagenetic analysis of bacterial profiles was performed via amplicon sequencing that covered the V3-V4 fragment of the 16S rRNA gene. Next-generation sequencing was applied to test the specificity of our WOLBSL and WOLBSR primers [32] to *Wolbachia* 16S rRNA sequences in the microbiome community. In addition, to test whether *Wolbachia* can also be detected in the same samples using commercial primers, a simultaneous amplification was performed using 341F (forward: 5′–CCTACGGGNGGCWGCAG–3′) and 785R (reverse: 5′–GACTACHVGGGTATCTAATCC–3′) primers [106], indicated by Klindworth et al. [107] as the most suitable for Illumina sequencing of target gene regions (Figure 5). In both cases, length filter (assembled read) 400 bp ≤ good amplicon sequences ≤ 500 bp have been applied.

The PCR amplification of the bacterial fragment of the 16S rRNA gene using WOLBSL and WOLBSR primers was performed under conditions described by Mioduchowska et al. [32]. In turn, amplification of the 16S rRNA gene fragment using 341F and 785R primers were performed in 20 µL volume containing 0.8× JumpStart Taq ReadyMix (1 U of JumpStart Taq DNA polymerase, 4 mM Tris-HCl, 20 mM KCl, 0.6 mM MgCl_2_, and 0.08 mM of dNTP; Sigma-Aldrich, Germany), 0.4 µM of 341F and 785R primers and about 5 ng of DNA. The 16S rRNA gene fragment was amplified under the following conditions: initial denaturation at 95 °C for 3 min followed by 25 cycles of 95 °C for 30 s, 55 °C for 30 s and 72 °C for 30 s and ending with 72 °C for 5 min. The PCR products were separated in 1% agarose gel in a 1× SB buffer and it was visualized using Midori Green Advance DNA Stain (Genetics) under UV light (Vilber Lourmat V01 7107). 

### 4.4. Generation of the 16S rRNA Amplicon Library and Taxonomic Classification

We decided to analyze samples of taxa for which we detected *Wolbachia* using our and *Wolbachia*-specific primers or for which poor Sanger sequences were obtained using the designed primers (different bacterial species from one sample were amplified simultaneously) despite good quality PCR products. All samples for which non-target *Wolbachia* sequences have been obtained, e.g., uncultured bacteria [32], were removed from the NGS analysis.

Indexing PCR reactions, which were performed using Q5 Hot Start High-Fidelity 2× Master Mix, was the next step prior to NGS. Reaction conditions were used according to the manufacturer’s recommendations. All PCR products obtained using commercial primers passed the final Quality Control (QC). Libraries of appropriate quality were obtained for one species of Bivalvia, i.e., *D. polymorpha* and three taxa of Crustacea, i.e., *A. salina*, *A. parthenogenetica*, and *Eulimnadia* sp. when primers designed by us were used. However, library concentrations of the remaining samples (Crustacea: *Chydorus* sp., *B. schaefferi*, *T. cancriformis*; Bivalvia: *U. crassus*; Tardigrada: *Pam. experimentalis* and *Mac. basiatus*) were below the detection limit, despite the good quality of the PCR reaction products. Paired-end (PE) sequencing was performed with an Illumina MiSeq platform (Genomed, Poland).

Automatic preliminary data analysis was performed using a MiSeq apparatus and MiSeq Reporter (MSR) v2.6 (https://www.illumina.com/systems/sequencing-platforms/miseq/products-services/miseq-reporter.html; URL accessed on 16 March 2018.

Taxonomic classification of the bacterial 16S rRNA gene was performed using QIIME 2 [108], based on the GreenGenes v13.8 reference sequence database [109]. The analysis consisted of the following stages:

(1) removing adapter sequences using the cutadapt program [110], 

(2) analysis of read quality and removal of low-quality sequences (quality <20), using cutadapt program [110], 

(3a) commercial primers: paired sequences were joined using the fastq-join algorithm [111], 

(3b) designed primers: due to the amplicon size >450 bp, paired readings were treated as individual reads and not as pairs, 

(4a) commercial primers: for clustering based on the selected GreenGenes v13.8 reference sequence database, the uclust algorithm [112] was used; chimeric sequences were removed using the ChimeraSlayer algorithm [113] in 16S rRNA analysis. The taxonomy to the selected reference sequence database was assigned using the uclust algorithm with a sequence similarity limit of 97%,

(4b) designed primers: clustering and taxonomy assignment were carried out based on the GreenGenes v13.8 reference sequence database, without the possibility of forming new clusters (closed-reference OTU picking), which allowed to study two regions simultaneously (i.e., read 1 and read 2 as separate regions); the taxonomy to the selected reference sequence database was assigned using the uclust algorithm with a sequence similarity limit of 97%,

(5) visualization of the microbiome profile obtained as a result of 16S rRNA fragment amplification using two pairs of primers was performed in Geneious 2022.0.2 (http://www.geneious.com, URL accessed on 18 January 2022), and

(6) the comparison of bacterial community structure, i.e., the Venn diagram; OTUs common to the microbiome from all stations were visualized using R 4.0.3 [114] with the VennDiagram package.

### 4.5. Python Script to Identify the Target Wolbachia Infection in the Microbiome Community

**Figure 6:**
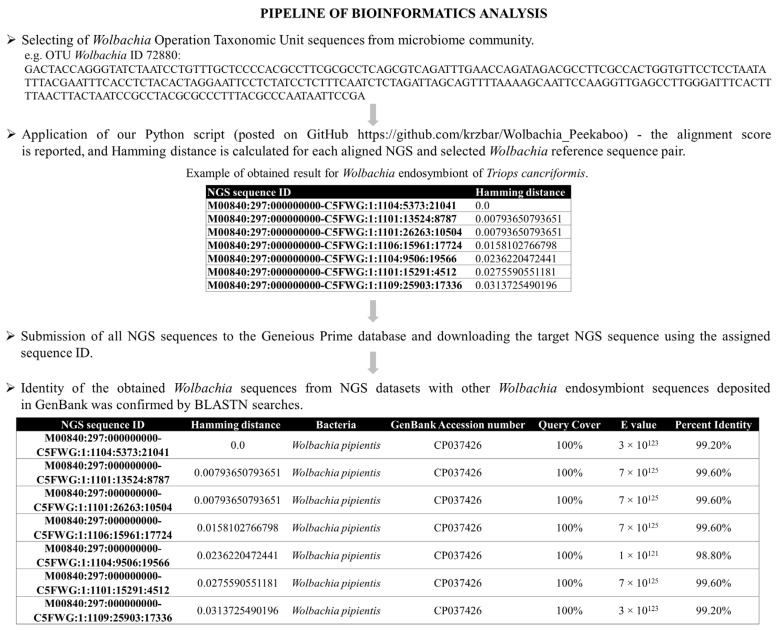
Application workflow of Python script.

We wrote a Python script to detect *Wolbachia* sequences from the microbiome community based on Hamming distance (pairwise distance) values. Hamming distance values were calculated using the *Wolbachia* operational taxonomic unit (OTU) detected in our study as the reference sequence (details in Figure 6).

In principle, our script first reads in all available fastq files in its working directory. It is assumed that each amplified sample is present in two files (from reverse and forward primers separately), following each other alphabetically. Then, it reads the target *Wolbachia* sequences. For each pair of target and amplified sample sequence, our script calculates a local alignment score and subsequently the Hamming distance (pairwise distance) score between the two aligned sequences. The local alignment is adjusted using Biopython’s pairwise2.align.localxx (+1 score for identity, and penalty equal to 0 for mismatch or gap) implementation of the dynamic programming local alignment algorithm. The local alignment is required, as the Hamming distance is defined for sequences of equal length. When calculating this pairwise distance, we assumed 0 for the match, 1 for the mismatch, and the gaps were treated as mismatches. Afterwards, the amplified sample sequences are returned and sorted according to their Hamming distance (ascending, i.e., more similar ones with smaller distances) and according to their local alignment scores (descending, i.e., more similar ones with higher scores). The returned outputs are two .csv files (per pair of fastq files) with lists of amplified sample sequence identifiers and their scores (Python script has been posted on GitHub (17 May 2023): https://github.com/krzbar/Wolbachia_Peekaboo). The bioinformatics pipeline is shown in Figure 6.

### 4.6. Phylogeny of Identified Wolbachia Strains

Different fragments of the 16S rRNA gene of the genus *Wolbachia* are deposited in the GenBank database due to the application of various primers for the amplification. Therefore, the data for H and L supergroups of the *Wolbachia* strains could not be used in phylogenetic analysis. We downloaded the data from GenBank on 16 supergroups of *Wolbachia* strains from different invertebrate hosts species and aligned them in our dataset. The quality of all the sequences obtained was then checked and trimmed to the same length in BioEdit v. 7.2.5 [102]. The alignment was conducted in CLUSTAL W [115] with default settings. Recombination between strains was detected using the φ test implemented in SplitsTree4 [116]. The φ test did not find statistically significant evidence for recombination (p = 0.6002). Differentiation between the obtained *Wolbachia* supergroups was derived from a phylogenetic analysis and uncorrected p-distances calculated in MEGA X [117].

The phylogeny of the 16S rRNA *Wolbachia* sequences obtained from the microbiome community of freshwater invertebrate hosts and sequences representing different *Wolbachia* phylogenetic supergroups were tested using Bayesian inference (BI) analyses using MrBayes v.3.2.6 [118] implemented in the Geneious 2022.0.2. The *Ehrlichia chaffeensis* Anderson, Dawson, Jones and Wilson, 1991 ([119], order Rickettsiales) sequence was added to the analysis as an outgroup. We also reconstructed the phylogenetic relationships between all *Wolbachia* sequences identified in Tardigrada until now: (i) twelve sequences from our study; (ii) three *Wolbachia* OTUs from Mioduchowska et al. [34] and (iii) one *Wolbachia* OTU from Tibbs-Cortes et al. [36]. As an outgroup, we applied all Rickettsiales OTUs described by Mioduchowska et al. [34] and Tibbs-Cortes et al. [36]. The most appropriate sequence evolution model was determined via the jModelTest [120] for sequence evolution modeling, and both the Bayesian Inference Criterion (BIC) and Akaike Information Criterion (AIC) most highly supported the Hasegawa, Kishino, and Yano (HKY model with proportion of invariable unchanging sites) model. The following settings were applied: the chain length—1,100,000, heated chains—4, subsampling frequency—200, burn-in length—110,000 and heated chain temperature—0.2. The generated phylogenetic trees were viewed and visualized using Inkscape 1.0 (4035a4fb49, 27 January 2023) [121]. 

## Figures and Tables

**Table 1:** List of *Wolbachia* supergroups (literature overview until our study).

*Wolbachia* Supergroup	Host	Host–*Wolbachia* Association	Reference
**A**	**Arthropods**: - **insects (Insecta)**: flies (Diptera), butterflies and moths (Lepidoptera), beetles (Coleoptera), wasps and bees (Hymenoptera), bugs, aphids, whiteflies and psyllids (Hemiptera) and other - **spiders (Araneae)**, e.g., *Nurscia* sp. (Titanoecidae), *Telema* cave spiders (Telemidae) - **isopods (Isopoda)**, e.g., *Burmoniscus* sp. (Oniscidea)	mutualism, reproductive parasitism	[8,9,10,11]
**B**	**Arthropods**: - **insects (Insecta)**: butterflies and moths (Lepidoptera), leafhoppers, whiteflies and aphids (Hemiptera), wasps (Hymenoptera), beetles (Coleoptera), flies and mosquitoes (Diptera) and other - **spiders (Aranae)**, e.g., *Hylyphantes* sp. (Linyphiidae) - **isopods (Isopoda)**, e.g., woodlouse (Oniscidea) - **mites (Acari)**: spider mites (Tetranychidae)	mutualism, reproductive parasitism	[8,9,11]
**C**	**Filarial nematodes (Nematoda: Filariidae)**	mutualism	[12,13]
**D**	**Filarial nematodes (Nematoda: Filariidae)**	mutualism	[12,14]
**E**	**Arthropods**: - **springtails (Collembola)** - **mites (Acari)**: oribatid mites (Oribatida)	mutualism, reproductive parasitism or undetermined	[15,16,17,18]
**F**	**Arthropods**: - **insects (Insecta)**: bugs (Hemiptera), parasitise lice (Phthiraptera), termites (Isoptera) and others - **scorpions (Scorpiones)**: burrowing scorpions *Opistophthalmus* sp. (Scorpionidae) - **isopods (Isopoda)**, e.g., the Neotropical isopod *Neotroponiscus* sp. (Oniscidea) **Filarial nematodes (Nematoda: Filariidae)**	mutualism, reproductive parasitism	[9,12,19,20]
**H**	**Arthropods** - **insects (Insecta)**: termites (Isoptera)	undetermined	[21]
**I**	**Arthropods** - **insects (Insecta)**: fleas (Siphonaptera)	undetermined	[22,23]
**J**	**Filarial nematodes**	undetermined	[12]
**K**	**Arthropods** - **mites (Acari)**: spider mites (Tetranychidae)	undetermined	[23]
**L**	**Plant nematodes**	undetermined	[24]
**M**	**Arthropods** - **insects (Insecta)**: aphids (Hemiptera)	undetermined	[25]
**N**	**Arthropods** - **insects (Insecta)**: aphids (Hemiptera)	undetermined	[25]
**O**	**Arthropods** - **insects (Insecta)**: *Bemisia tabaci* (Gennadius) [26] whiteflies (Hemiptera)	undetermined	[27]
**P**	**Arthropods** - **mites (Acari)**: syringophilid mites (Cheyletoidea)	undetermined	[28]
**Q**	**Arthropods** - **mites (Acari)**: syringophilid mites (Cheyletoidea)	undetermined	[28]
**S**	**Arthropods** - **pseudoscorpions**	undetermined	[12]
**T**	**Arthropods** - **insects (Insecta)**: *Cimex hemipterus* (Fabricius) [29] (Hemiptera)	undetermined	[30]
**U**	**Arthropods** - **mites (Acari)**: bat mites *Spinturnix* sp. (Spinturnicidae)	undetermined	[31]

**Table 3:** Summary of *Wolbachia* infection prevalence. Symbols and abbreviations: *—isolates for which we also obtained using Sanger sequences; NA—not available.

Phylum	Species (Isolate ID)	The Number of Obtained Forward and Reverse Sequences of *Wolbachia*/the Number of *Wolbachia* OTUs (% of *Wolbachia* Sequences in Microbiome Community)		p-Distance Value between Our and the Most Similar *Wolbachia* Sequences Deposited in GenBank (Accession Numbers Provided in Brackets) Generated Using Our Python Script
		Result Obtained Using Commercial 341F/785R Primers	Result Obtained Using Our Designed WOLBSL/WOLBSR Primers	
**ARTHROPODA**	*Artemia salina* (AS)	NA	31/2 (0.12)	p-distance: 0.00–0.14 (CP037426, GQ167636, DQ235279)
	*Artemia* *parthenogenetica* (AP)	NA	28/4 (0.01)	p-distance: 0.01–0.14 (CP037426, JX182385, GQ167636, EF417899)
	*Branchipus schaefferi* (PA)	NA	* [32]	* [32]
	*Branchipus schaefferi* (SRB1)	NA	32/3 (26.23)	p-distance: 0.01–0.04 (CP037426, MT588740)
	*Chydorus* sp. (ALTAJ2)	61/4 (0.05)	2/2 (0.35)	p-distance: 0.01 (CP042445)
	*Eulimnadia* sp. (CON)	NA	56/6 * (present study) (0.02)	p-distance: 0.00–0.19 (MT588740, AJ306314, GQ167636, CP037426, KT319089), * (present study, MZ901361)
	*Streptocephalus cafer* (SC)	NA	* [32]	* [32]
	*Triops cancriformis* (TCO)	NA	113/4 (38.97)	p-distance: 0.00–0.15 (CP037426, GU236947, EF417899)
**MOLLUSCA**	*Unio crassus* (population C3Gf)	NA	10/2, * [35] (8.33)	p-distance: 0.00–0.02 (MT588740), * [35]
	*Unio crassus* (population P3Nf)	NA	NA	NA
	*Dreissena polymorpha* (RAC)	NA	40/4 (0.01)	p-distance: 0.00–0.09 (MT588740, DQ235279, CP042904, AY157501, KT319089)
**TARDIGRADA**	*Paramacrobiotus* *experimentalis* (population MAD-TAR9)	NA	2/1 (14.29)	p-distance: 0.02 (MT588740)
	*Paramacrobiotus* *experimentalis* (population MAD-TAR11)	NA	16/2 (42.11)	p-distance: 0.01–0.07 (MT588740)
	*Macrobiotus basiatus* (8aUSA)	2/1 (0.001)	22/2 (16.92)	p-distance: 0.00–0.07 (CP042445)

## Data Availability

Not applicable.

## Supplementary Materials

The supporting information can be downloaded at: https://www.mdpi.com/article/10.3390/ijms24119400/s1.
